# Complete Genome Sequence of Cluster J Mycobacteriophage Superphikiman

**DOI:** 10.1128/genomeA.01538-17

**Published:** 2018-02-01

**Authors:** Pratik Pradhan, Sprikena Nako, Trinh Tran, Lavanya S. Aluri, Dharman Anandarajan, Niteesha Betini, Shivangi D. Bhatt, Swetha Chengalvala, Nicole E. Cox, Bela P. Delvadia, Aishwary S. Desai, Andrew M. Devaney, Brenna K. Doyle, Arden O. Edgerton, Matthew C. Erlich, Kevin C. Fitzpatrick, Esha A. Gajjar, Anjali Ganguly, Ramnik S. Gill, Pauline M. Good, Nishtha Gupta, Leila M. Haddad, Esther J. Han, Shelby Jain, Andrew Jiang, Andrew D. Jurgielewicz, Devneet K. Kainth, Jawhara M. Karam, Mallika Kodavatiganti, Sinja J. Kriete, Catherine E. MacDonald, Josh P. Maret, Ashley E. Mathew, Maanasa Natrajan, Nusrat M. Nishu, Nirali Patel, Pooja D. Patel, Shivani Patel, Kaustav Patra, Karima K. Rai, Arghyadeep Sarkar, Priyanka Shah, Ravi K. Tata, Andrew H. Tawfik, Bhavya T. Thuremella, Justina Toma, Shika Veera, Vamsee K. Vemulapalli, Trevor V. Vidas, Katy S. Vieira, Gayathri Vijayakumar, Tru A. Walor, Clara R. White, Brianna M. Wong, Shu L. Zhao, David W. Bollivar, Matthew T. McDonald, Ritu R. Dalia, Kevin P. W. Smith, Joy L. Little, Susan M. R. Gurney

**Affiliations:** a Department of Biology, Drexel University, Philadelphia, Pennsylvania, USA; b Department of Biological Sciences, Illinois Wesleyan University, Bloomington, Illinois, USA; c Department of Biological Sciences, North Carolina State University, Raleigh, North Carolina, USA

## Abstract

Mycobacteriophage Superphikiman is a cluster J bacteriophage which was isolated from soil collected in Philadelphia, PA. Superphikiman has a 109,799-bp genome with 239 predicted genes, including 2 tRNA genes.

## GENOME ANNOUNCEMENT

Mycobacteriophages are viruses which are known to infect mycobacterial host species (1). Mycobacteriophage Superphikiman was isolated from soil collected on the Drexel University campus in Philadelphia, PA, USA, by using *Mycobacterium smegmatis* mc^2^155 as a host. Superphikiman was isolated using the direct isolation procedure at 37°C in the fall of 2015. Its plaques were clear and consistently measured 1 mm in diameter. Electron microscopy showed that this bacteriophage has a *Siphoviridae* morphology.

Superphikiman plaques were selected, purified, and amplified, and the DNA was extracted. The Superphikiman genome was sequenced with the Illumina MiSeq platform using 150-bp single-end reads and had an average coverage of 335×. The genome was then annotated using the following databases and software: DNA Master (http://cobamide2.bio.pitt.edu), Glimmer (2), GeneMark (3), Starterator, Phamerator (4), PhagesDB.org, NCBI BLAST and Conserved Domain Database at NCBI (5, 6), HHPRED (7), ARAGORN (8), tRNAscan-SE (9), and PECAAN (http://pecaan.kbrinsgd.org).

Superphikiman is a member of actinobacteriophage cluster J (10). The Superphikiman genome is 109,799 bp in length, with a 4-base 3′ single-stranded DNA extension (right end 5′-ATCC) and a 61% G+C content. After annotation, 239 genes were identified, with 21.3% of genes being assigned a function. Superphikiman was compared to other bacteriophage genomes by using NCBI Blast (https://blast.ncbi.nlm.nih.gov/) and was found to be most similar to bacteriophage Courthouse (GenBank accession number JN698997), with 99% identity over 97% coverage.

The Superphikiman genome is arranged canonically, with the structural genes and viral assembly genes on the leftmost 35 kbp of the genome. At the rightmost 15 kbp of the genome, there are consecutive small reverse genes. A similar arrangement is found in other cluster J phages, including Courthouse. The Superphikiman genome has a lysis cassette consisting of lysin A, lysin B, and holin genes. Between the lysis cassette and immunity cassette there are 32 short reverse genes, which were not found to have any specific functions. Given their position in the genome it is possible that these genes are involved in phage-host defense/counter defense systems. Other cluster J bacteriophages such as Courthouse have short reverse genes in this position; however, the number of genes present and the nucleotide sequences vary across this region (Phamerator.org). The immunity repressor in Superphikiman (gp191) is identical to the immunity repressor genes found in the cluster A1 bacteriophages Dynamix (gp75) (GenBank accession number AMD43071) and Abrogate (gp72) (GenBank accession number YP_009209461). The genome also contains the WhiB gene (gp126), which is shared by other cluster J bacteriophages but is not found in other phage clusters (Phamerator.org).

When the tRNA genes in Superphikiman were compared to those of three other cluster J phages (Courthouse, MiaZeal [GenBank accession number KM925136], and Ariel [GenBank accession number KM400683]), it was found that all four genomes contain 2 tRNA genes at approximately the same location in the genome and that these tRNA genes code for glycine and tyrosine. Other annotated cluster J bacteriophages (PhagesDB.org) have been found to contain tRNA genes for either glycine or tyrosine, or both, but not for other tRNA genes.

### Accession number(s).

This genome sequence has been deposited at GenBank under the accession number MF919534. The version described in this paper is the first version, MF919534.1.
